# Dissemination of *Escherichia coli* with CTX-M Type ESBL between Humans and Yellow-Legged Gulls in the South of France

**DOI:** 10.1371/journal.pone.0005958

**Published:** 2009-06-18

**Authors:** Jonas Bonnedahl, Mirva Drobni, Michel Gauthier-Clerc, Jorge Hernandez, Susanne Granholm, Yves Kayser, Åsa Melhus, Gunnar Kahlmeter, Jonas Waldenström, Anders Johansson, Björn Olsen

**Affiliations:** 1 Department of Medical Sciences, Section of Infectious Diseases, Uppsala University, Uppsala, Sweden; 2 Clinical Microbiology, Kalmar County Hospital, Kalmar, Sweden; 3 Centre de Recherche de la Tour du Valat, Le Sambuc, Arles, France; 4 Department of Clinical Microbiology, Infectious Diseases and Bacteriology, Umeå University, Umeå, Sweden; 5 Department of Medical Sciences, Section of Clinical Bacteriology, Uppsala University, Uppsala, Sweden; 6 Clinical Microbiology, Central Hospital, Växjö, Sweden; 7 School of Natural Sciences, Section of Zoonotic Ecology and Epidemiology, University of Kalmar, Kalmar, Sweden; Technical University Munich, Germany

## Abstract

Extended Spectrum β-Lactamase (ESBL) producing Enterobacteriaceae started to appear in the 1980s, and have since emerged as some of the most significant hospital-acquired infections with *Escherichia coli* and *Klebsiella* being main players. More than 100 different ESBL types have been described, the most widespread being the CTX-M β-lactamase enzymes (*bla*_CTX-M_ genes). This study focuses on the zoonotic dissemination of ESBL bacteria, mainly CTX-M type, in the southern coastal region of France. We found that the level of general antibiotic resistance in single randomly selected *E. coli* isolates from wild Yellow-legged Gulls in France was high. Nearly half the isolates (47,1%) carried resistance to one or more antibiotics (in a panel of six antibiotics), and resistance to tetracycline, ampicillin and streptomycin was most widespread. In an ESBL selective screen, 9,4% of the gulls carried ESBL producing bacteria and notably, 6% of the gulls carried bacteria harboring CTX-M-1 group of ESBL enzymes, a recently introduced and yet the most common clinical CTX-M group in France. Multi locus sequence type and phylogenetic group designations were established for the ESBL isolates, revealing that birds and humans share *E. coli* populations. Several ESBL producing *E. coli* isolated from birds were identical to or clustered with isolates with human origin. Hence, wild birds pick up *E. coli* of human origin, and with human resistance traits, and may accordingly also act as an environmental reservoir and melting pot of bacterial resistance with a potential to re-infect human populations.

## Introduction

The levels of antibiotic resistance in *Escherichia coli* have reached a point where they pose severe clinical challenges to humans. *E. coli* is frequently used for studies of resistance status and development, and displays a relatively rapid transfer of resistance between strains, particularly if plasmid-borne [1], [2]. It is also a major player in the spread of the recently emerged resistance to β-lactam antibiotics through the action of CTX-M β-lactamases, enzymes that have their origin in chromosomally located genes of other members of the Enterobacteriaceae family, i.e. *Kluyvera* sp. and possibly others [1]. Enterobacteriaceae that produce extended spectrum β-lactamases (ESBLs) started to appear in the 1980s, and have since emerged as some of the most significant hospital-acquired infections [1]. ESBL-producing bacteria, mainly *E. coli* and *Klebsiella,* are rapidly increasing among human isolates and today more than 100 different ESBL types have been described, with the most widespread type being the CTX-M β-lactamase enzymes (hereafter CTX-M) encoded by the *bla*_CTX-M_ gene. The first CTX-M was characterized in 1989, but today CTX-M have a large geographical distribution [1], [3]. In France reports of CTX-M emergence arose with the new century and several outbreaks were described in northern France 2002–2003. In southern France, other types of ESBL accounted for >90% of the ESBLs in a 2002–2003 survey. In this region, CTX-M started to appear later but have from 2004 been identified in several hospitals [3]. The CTX-M has also spread to other microorganisms, including community species such as *Salmonella*, *Shigella* and *Vibrio cholera* [3].

Migrating birds seem to act as transporters, or as reservoirs, of resistant bacteria and could therefore have an important epidemiological role in the dissemination of resistance, as well as being mirrors of the spectrum of pathogenic micro-organisms present in humans. A human-bird transfer of *E. coli* has been shown by the finding of a higher *E. coli* presence in wild birds with human association than in birds lacking human contacts [4]. Furthermore, although animals isolated from human activities previously displayed lower levels of resistant bacteria [5], more recent studies now show the presence of resistant *E. coli* in wild birds regardless of isolation. We saw *e.g.* that *E. coli* in Arctic birds sampled at the Siberian tundra carried antibiotic resistance, an indication of worldwide resistance dissemination [6]. In Maryland, US, migratory geese were carriers of resistant *E. coli,* as were Black-headed Gulls in the Czech Republic [7], [8]. *E. coli* harboring β -lactamases of CTX-M, TEM and SHV type have previously been detected in birds of prey and seagulls in Portugal [9], [10].

**Figure 1:**
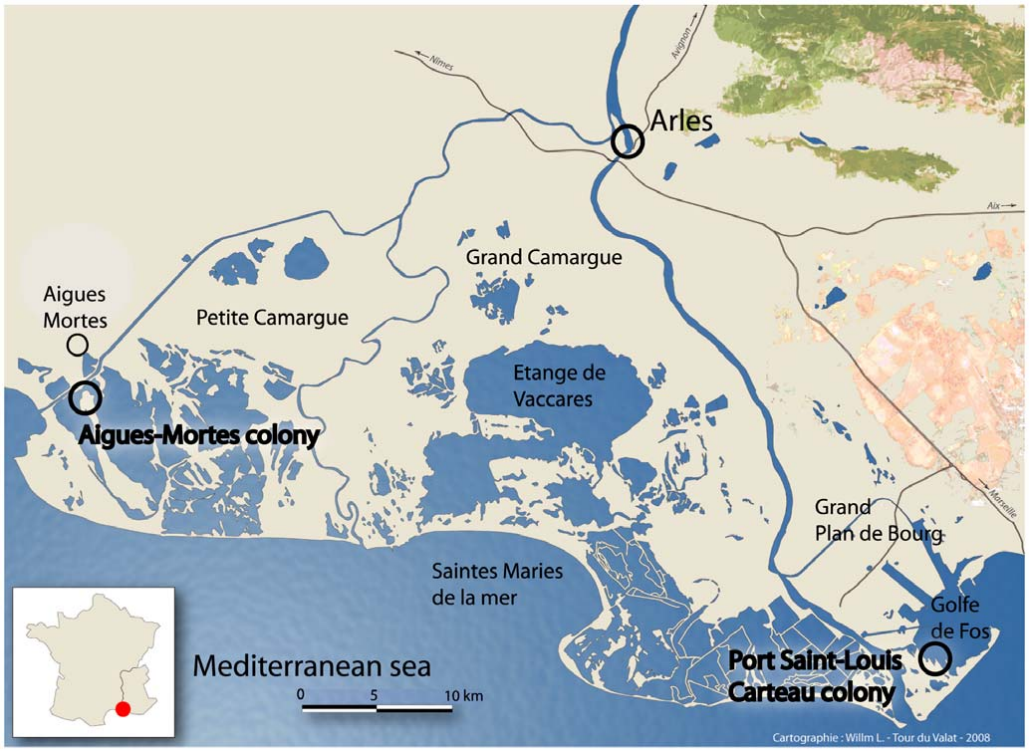
Map shows location of Yellow-legged Gull populations in the study. Port Saint-Louis Carteau colony feeds mainly at the Marseille city dump, and Aigues-Mortes colony mainly offshore.

We investigated the presence of antibiotic resistance, with special focus on CTX-M ESBL bacteria, in Yellow-legged Gulls (*Larus michahellis)* in two populations in Camargue, southern France (Figure 1). This species belongs to the Herring Gull-complex, a set of large size gulls including several species present in the northern hemisphere. Many of these gulls, including the Yellow-legged Gull, have increased in numbers and distribution due to their custom of living in proximity to humans. They are often opportunistic marine feeders, seeking their food (fish, bivalves, mollusks, eggs, birds etc) along the shoreline or offshore, but also readily utilizing the food sources provided by humans, especially garbage. The two Yellow-legged Gull populations are located in a region with a dense human population, and were chosen in order to take into account any variations in resistance patterns resulting from differences in the availability of human-associated food sources. The gulls breeding at Port Saint-Louis Carteau colony feed mainly at Marseille city dump, whereas the gulls at Aigues-Mortes colony feed mainly offshore (Figure 1). Our results show that resistance levels of randomly selected *E. coli* (one strain from each sample) are high when measured against a panel of six antibiotics, and that wild birds in this region carry high levels of ESBL producing bacteria displaying genotypes highly related with French (human) clinical samples. Further, ESBL producing *E. coli* from these birds shared phylogenetic diversity with human *E. coli*, coherent with that these bacteria are established zoonotic agents.

## Results

### Antibiotic resistance-levels in *E. coli* from Yellow-legged Gulls

In order to determine a general picture of resistance levels in Yellow-legged Gulls we tested one randomly selected *E. coli* isolate per sample, to a panel of six antibiotics. Nearly half (47.1%; 72/153 isolates) of the *E. coli* isolates were resistant to at least one antibiotic, and there was no difference in overall resistance rates between the two gull colonies (33/75 *E. coli* isolates from “city dump” colony and 39/78 isolates from “off shore” colony). Resistance to two or more antimicrobial agents was widespread, and found in approximately 1/3 of the isolates. The most widespread resistance phenotypes were those resistant to tetracycline, ampicillin and streptomycin. Resistances to the remaining compounds were less frequent (Table 1). Four strains displayed resistance to cefadroxil (Table 1), an indication of potential ESBL carriage.

**Table 1:** Antibiotic resistance of single randomly selected *E. coli* isolates from bird fecal samples.

Antibiotic	Colony by feeding site	
	“City Dump” (n = 75)	“Offshore” (n = 78)
Tetracycline	27	32
Ampicillin	19	19
Streptomycin	15	14
Chloramphenicol	2	8
Nalidixic acid	2	2
Cefadroxil^1^	4	0

1 Cefadroxil resembles cefalexin and is used to screen for ESBLs as part of the recommendations by the SRGA.

### Selective screen for ESBL producing bacteria

ESBL producing bacteria were initially identified by phenotypic tests. 17 isolates (16 *E. coli* and one *Enterobacter cloacae*, all from separate samples) were found that exhibited disc diffusion synergy test and cefepime MIC concordant with ESBL production, as well as growth on cefpodoxime containing chromID™ ESBL plates. The presence of *bla*_CTX-M_, *bla*_TEM_ and *bla*_SHV_ was then determined by PCR. Ten isolates were confirmed to harbor CTX-M-1-group enzymes, and sequencing defined genotypes to 9 isolates (including the *E. cloacae*) with *bla*_CTX-M-1_ and one with *bla*_CTX-M-15_ (Table 2). *bla*_TEM_ was found in combination with *bla*_CTX-M_ in two isolates and with *bla*_SHV_ in two isolates, and separately in five isolates. All isolates, containing any of the *bla*-genotypes, displayed ESBL positive results in Etests with cefotaxime, ceftazidime and cefepime combined with clavulanic acid inhibition. Three of the four fecal samples containing cefadroxil resistant, randomly selected, *E. coli* also generated ESBL producing bacteria in the ESBL selective screen (*E. coli* with sample ID 63638, 63654, and 63725; Table 2).

**Table 2:** Genotypic analyses of phenotypically positive ESBL producing bacteria.

	Isolate ID	Species	*bla*-genotype		
			CTX-M^a^	TEM^c^	SHV^b^
“Offshore”	63541	*E. coli*	−1		
	63546	*E. coli*		+	+
	63560	*E. coli*		+	
	63562	*E. coli*	−15	+	
	63574	*E. coli*		+	+
“City Dump”	63606	*E. coli*	−1		
	63633	*E. coli*	−1		
	63638	*E. coli*	−1		
	63652	*E. coli*		+	
	63654	*E. coli*	−1		
	63657	*E. coli*		+	
	63659	*E. coli*	−1		
	63686	*E.cloacae*	−1		
	63688	*E. coli*	−1	+	
	63706	*E. coli*		+	
	63725	*E. coli*	−1		
	63727	*E. coli*		+	

a *bla*_CTX-M_ positive isolates were sequenced for specific genotypes, see methods and materials.

b + indicates presence of *bla*_TEM_ and *bla*_SHV_ genes as detected by real-time PCR, see methods and materials.

### Multi locus sequence typing and phylogenetic group determination of the ESBL producing isolates

**Figure 2:**
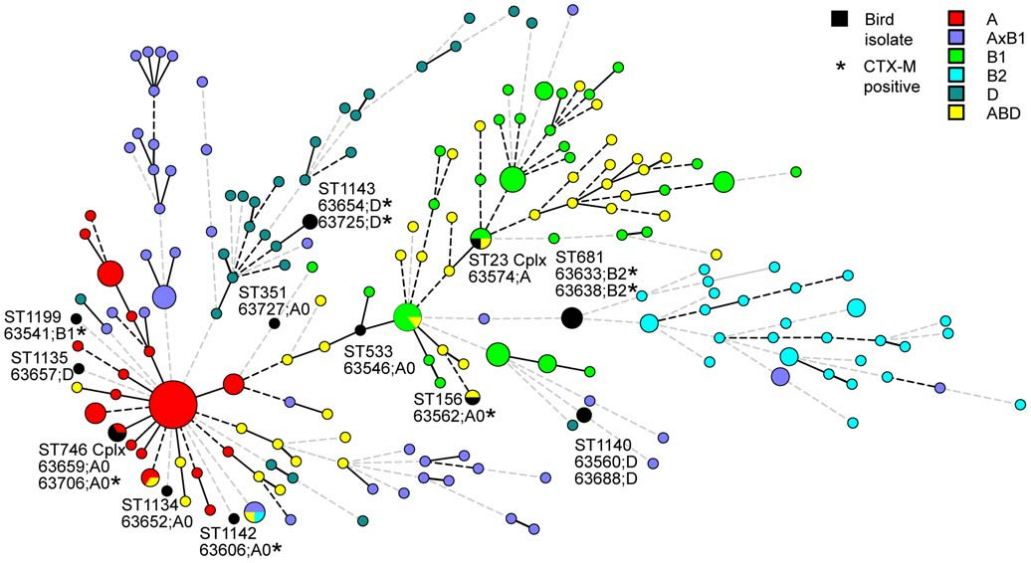
Distribution of 16 ESBL producing *E. coli* isolates within a minimum spanning tree representing 273 previously reported STs (dots) from a collection of 459 diverse *E. coli* isolates. The tree is based on the degree of allele sharing by MLST analysis. Clonal complexes composed of at least three ST members are indicated by dots proportional in size to the number of STs within them. STs, isolate designations, phylogenetic group, and distribution of CTX-M genes of ESBL producing *E. coli* from birds are indicated (black dots). Uniformly colored dots indicate a shared phylogenetic group. Thick lines connecting pairs of STs indicate that they share six (thick lines) or five (thin lines) alleles. Dotted connecting lines represent less allele sharing.

Based on sequencing of 3423 nucleotides at seven house-keeping genes, the sixteen ESBL-producing *E. coli* were assigned to 13 different sequence types (STs) (Table 3). Nine isolates were assigned to seven novel STs, *i.e.*, allelic profiles that were not found in the multi locus sequence typing (MLST) database; STX1 (1 isolate), STX2 (2 isolates), STX3 (1 isolates), STX4 (1 isolate), STX5 (2 isolates), STX6 (1 isolate), and STX7 (1 isolate) (Table 3). Seven isolates were assigned to six previously reported STs; single isolates to ST156, ST90, ST351 or ST746, respectively, and two isolates to ST681. The 13 STs representing the 16 ESBL-producing *E. coli* isolates were compared with 273 STs representing full global diversity of the species (Figure 2) [11]. It was apparent that the 16 ESBL isolates covered a broad range of genetic diversity. Consequently, a large fraction of the currently known *E. coli* genetic diversity was present among the ESBL-producing isolates and they were not of recent common ancestry. Specifically, only those isolates that are found in a clonal complex or share an ST are likely to have a recent common ancestry, *i.e.*, isolates in STX2, in STX5, or in ST681.

**Table 3:** MLST and phylogenetic group assignments for ESBL-producing *E. coli* isolates.

Isolate ID	ST	Clonal complex	Phylogenetic group^a^
63541	ST1199	–	B1
63546	ST533	–	A0^b^
63560	ST1140	–	D
63562	ST156	ST156	A0
63574	ST90	ST23	A
63606	ST1142	–	A0
63633	ST681	–	B2
63638	ST681	–	B2
63652	ST1134	–	A0
63654	ST1143	–	D
63657	ST1135	–	D
63659	ST1144	ST746	A0
63688	ST1140	–	D
63706	ST746	ST746	A0
63725	ST1143	–	D
63727	ST351	–	A0

a Phylogentic group as determined by the triplex PCR method (ref Clermont).

b A0 denotes that all three DNA targets of the triplex PCR failed to amplify.

According to the classification method by Clermont et al. [12], later modified by Escobar-Páramo et al. by addition of subgroups [13], seven isolates belonged to phylogenetic group A0, five to group D, one to group B1, two to group B2, and one to group A (Table 3). The seven isolates classified as A0 were not closely related as determined by MLST, they were frequently found interleaved among isolates of other phylogroups and were widely dispersed in the minimum spanning tree (Figure 2). For other phylogenetic groups, there was some correspondence with the position of a corresponding ST in the minimum spanning tree, e.g. isolates of STX5 shared phylogroup D with other neighbor isolates and ST681 shared the B2 phylogroup with its neighbors (Figure 2). Phylogroup assignments within STs were consistent, isolates assigned to the same ST always shared phylogenetic group. The *bla*_CTX-M_ harboring *E. coli* were widely dispersed out in the minimum spanning tree showing that these resistance genes are present across the full *E. coli* genetic diversity.

## Discussion

In the Mediterranean region, including France, the usage of antibiotics in human and veterinary medicine is at a comparatively high level [14], and consequently we found a high general level of antibiotic resistance in *E. coli* from Yellow-legged Gulls in the Marseille region. We believe that the influence of antibiotics in the region has been such that wild bird populations with varied feeding habitats carry organisms displaying comparable resistance levels. In fact, the resistance patterns are similar, with high level resistance especially to tetracycline, streptomycin and ampicillin. These are common resistance types in domesticated and wild animals in contact with human activities [2], [9].

Of 17 (9.4%) ESBL type β-lactam resistant isolates, all 10 CTX-M type isolates (nine *E. coli*, one *E. cloacae*) belonged to the CTX-M-1 group, specifically nine of *bla*_CTX-M-1_ (including the *E. cloacae*) genotype and one *bla*_CTX-M-15_. This is consistent with French clinical (human) isolates, where the CTX-M-1 group is also the major type [15], and both *bla*_CTX-M-1_ and *bla*_CTX-M-15_ genotypes have been isolated [1]. One isolate was *E. cloacae*, often a clinically relevant species as well [15], [16]. Nine *bla*_TEM_ and two *bla*_SHV_ harboring isolates were present, all positive in phenotypic tests for ESBL. Two isolates included both *bla*_CTX-M_ and *bla*_TEM_, and two both *bla*_TEM_ and *bla*_SHV_.

We found that fecal samples from gull colonies contained a high prevalence of *E. coli* with antibiotic resistance traits and that ESBL producing isolates exhibited great genetic diversity. This suggests that dense bird colonies, such as the Yellow-legged Gulls in our study, might serve as melting pots for creation of new *E. coli* variants carrying antibiotic resistance, or alternatively, that these birds are remarkably effective samplers of genetically diverse ESBL-carrying *E. coli* from other sources, e.g. humans. The distribution pattern of ESBL producing isolates within the genetic structure of *E. coli* shows that not only a few sporadic imports of specific clones carrying antibiotic resistance has occurred. Regardless of where the ESBL-producing *E. coli* have evolved originally, the feeding habit of gulls creates opportunities for antibiotic resistance genes to be mixed with many different *E. coli* genetic backgrounds.

We noted a lack of association between phylogenetic group and the allele based population structure analysis by MLST (Figure 2). Our findings are consistent with recent findings of other workers. Gordon et al. found that *E. coli* isolates that fail to produce any PCR amplicon at the three genes targeted in the triplex PCR assay (group A0 isolates [13]) are genetically heterogeneous [17]. Accordingly, our seven A0 isolates were genetically diverse illustrating that the A0 phylogenetic group assignment is a poor predictor of genetic relatedness.

Isolates assigned to the same STs or ST complex are likely to have a common recent ancestor. Our findings in bird samples of *E. coli* of STs that have been recovered from humans might therefore indicate transmission between man and birds. We found an ESBL-producing member of the ST23 clonal complex (Figure 2), which has previously been reported for several pathogenic *E. coli* isolates of human origin. Isolates of the ST23 clonal complex were reported from urinary tract infections in California, Toulouse in France, and from the northwest of England [18], [19]. The isolate from England was, similar to our bird isolate, resistant to expanded-spectrum cephalosporins. Similarly, we recovered an ESBL producing *E. coli* of ST533, an ST previously reported for an isolate from a human urinary tract infection in Brazil and exhibiting *bla*_tem-1_ and *bla*_CTX-M-2_ genes. This data together with data from other studies support the fact that *E. coli* with or without antibiotic resistance traits are transmitted between humans and birds [20], [21].

To summarize, we believe that a high general resistance level in Yellow-legged Gulls in the south of France is a result of long-time direct or indirect exposure to human activities in an area with relatively high antibiotic pressure. The presence of ESBL producing Enterobacteriaceae is high (9.4%) and in particular the proportion of the CTX-M-1 group (6%), which in this region is a very recently emerged and rapidly increasing ESBL type. The fact that ESBL producing bacteria are phylogenetically dispersed among *E. coli* isolates of human clinical and animal origin, underlines our hypothesis that these bacteria are both zoonotic and anthropogenic in their behavior, as are the resistance types found in them. Plasmid-borne resistance, such as CTX-M type ESBL, is obviously rapidly disseminating since it is already present in high levels in this type of animal reservoir that the birds represent. Hence, our data together with other recent studies indicates that birds could act as important environmental bio-indicators, and reservoirs, of medically important pathogens and resistance genes, as well as a potential melting pot for the development of new resistance types.

## Materials and Methods

### Collecting of fecal samples

90 samples per colony were collected from juvenile non-fledged birds. Each sample consisted of a cotton swab that had been rotated in the cloacae of a bird to obtain fecal material which was then inoculated into bacterial freeze medium (Luria broth; BD, Sparks, USA, phosphate buffered saline containing 0.45% Na-citrate, 0.1% MgSO_4_, 1% (NH_4_)_2_SO_4_, and 4,4% glycerol). After sampling in the field, the samples were transported to the laboratory and frozen at −70°C for later examination.

### Bacterial isolation and antibiotic resistance testing

Each sample was plated on Juhlin 32 agar [22] for isolation of putative *E. coli* isolates. *E. coli* identity was confirmed by biochemical testing. Susceptibility of one isolate per sample (with 75/90 isolated from the “city dump” colony and 78/90 from the “offshore” colony) was tested against a set of antibiotic agents: tetracycline, ampicillin, streptomycin, chloramphenicol, nalidixic acid and cefadroxil. These antibiotics were selected to represent commonly used agents in *E. coli* infections in human and veterinary medicine. Susceptibility was determined by antibiotic disk diffusion on Iso-Sensitest agar (Oxoid, Basingstoke, UK) in accordance with the recommendations of the Swedish Reference Group for Antibiotics (SRGA)[22], using *E. coli* ATCC 25922 as a reference strain (as in all assessments).

### Isolation of ESBL producing bacteria and phenotypic characterisation

For detection of ESBL producing bacteria, samples were plated onto on MacConkey agar (Oxoid, Basingstoke, UK) with two antibiotic discs containing cefotaxime (5 µg) and ceftazidime (10 µg), respectively. Isolates showing reduced susceptibility to one or both antibiotics were identified by biochemical testing. Phenotypic confirmation of ESBL-production was performed by disc diffusion synergy test according to SRGA [23].Cefepime MICs were determined using Etest (bioMérieux, Marcy l'Etoile, France) in isolates with a negative synergy test. ESBL production was also confirmed with ESBL Etest for cefotaxime, ceftazidime and cefepime including clavulanic acid (bioMérieux), and growth on cefpodoxime containing chromID™ ESBL plates (bioMérieux, Marcy l'Etoile, France).

### Genetic characterization of ESBL producing bacteria

Isolates with ESBL-production were further analyzed for presence of CTX-M, TEM and SHV type ESBL enzyme genes (*bla*_CTX-M_) using previously described methods [24], [25]. The *bla*_CTX-M_ gene was sequenced from CTX-M type positive isolates, using previously described primers [26] and GenScript BacReady PCR system (GenScript Corporation, Piscataway, NJ).

### Multilocus sequence typing of ESBL producing *E. coli* isolates

We used the MLST scheme developed by Wirth et al. [11](http://web.mpiib-berlin.mpg.de), utilizing seven housekeeping genes: adenylate cyclase (adk), fumarate hydratase (fumC), DNA gyrase (gyrB), isocitrate/isopropylmalate dehydrogenase (icd), malate dehydrogenase (mdh), adenylosuccinate dehydrogenase (purA) and ATP/GTP binding motif (recA). The PCR reactions were as follows; initial denaturation at 95°C for 2 min, 30 cycles of; denaturation at 95°C for 1 min; annealing at various temperatures for 1 min; elongation at 72°C for 2 min, and a final elongation at 72°C for 5 min. Reactions were performed using a T Gradient Thermocycler (Biometra GmbH, Goettinger, Germany). For sequencing reactions a commercially available sequencing kit was used (Big Dye® Terminator v3.1 cycle sequencing kit, Applied biosystem, Foster City, CA, USA) and analyzed in a 3730xl DNA Analyzer (Applied Biosystems, Foster City, CA, USA) according to manufacturers' instructions. Primers sequences and annealing temperatures can be found at http://web.mpiib-berlin.mpg.de. All sequence traces were imported, aligned, trimmed and quality controlled aided by functions of the software Bionumerics v.5.1 (Applied Maths NV, Sint-Martens-Latem, Belgium). Re-sequencing was performed if required and MLST allele designations were determined via the electronic MLST database at http://web.mpiib-berlin.mpg.de. Novel ST designations were provided by the curator of the database (Table 3).

### Allele-based population genetic analysis of ESBL producing *E. coli*

The genetic relationships of ESBL producing *E. coli* were assessed by calculations based on MLST allelic profiles for isolates and comparison with existing profiles in the MLST database. We used the minimum spanning tree algorithm implemented in Bionumerics v.5.1 (Applied Maths NV) with priority rules set at first link genotypes that have maximum numbers of single-locus variants and then maximal numbers of single-locus variants and double-locus variants. Sequence types (STs) were assigned members of clonal complex if they shared alleles in six out of seven house-keeping genes. We choose ST1 to ST278 of the MLST database as a comparative dataset for analysis of isolates characterized in this study. ST1 to ST278 represent the known diversity of *E. coli* in terms of geography, pathogenicity, human and animal origins. Four STs of the MLST database were excluded from analysis since representing extreme outgroup isolates (ST102, ST125, ST133) or a lack of a phylogentic group designation (ST103) [11].

### Phylogenic analysis of ESBL producing bacteria E. coli

Phylogenetic groups were determined using the triplex PCR method developed by Clermont et al. [12], and later modified by addition of subgroups by Escobar-Páramo et al [13], but with modified PCR conditions [27].
